# 非小细胞肺癌EGFR-TKI治疗临床获益后颅内转移机制研究进展

**DOI:** 10.3779/j.issn.1009-3419.2015.08.09

**Published:** 2015-08-20

**Authors:** 娟 蒋, 成平 胡

**Affiliations:** 410008 长沙，中南大学湘雅医院呼吸科 Department of Respiratory Medicine, Central South University Xiangya Hospital, Changsha 410008, China

**Keywords:** 表皮生长因子受体酪氨酸激酶抑制剂, 肺肿瘤, 颅内转移, EGFR-TKI, Lung neoplasms, Intracranial metastasis

## Abstract

表皮生长因子受体酪氨酸激酶抑制剂（epidermal growth factor receptor tyrosine kinase inhibitor, EGFR-TKI）广泛用于治疗*EGFR*突变阳性的非小细胞肺癌（non-small cell lung cancer, NSCLC）。然而，部分患者在接受EGFR-TKI治疗后、原发病灶稳定甚至缩小的同时，却出现了新发颅内转移灶或者原有颅内病灶进展，其机制未明。近年来多项研究表明，这种现象可能与EGFR-TKI的药物代谢动力学、NSCLC原发灶与转移灶的异质性、*EGFR*突变本身特质及患者生存期的延长有关。因此，本文就NSCLC患者在EGFR-TKI治疗临床获益后发生颅内转移的相关机制研究进展作一综述。

## Abstract (en)

Currently epidermal growth factor receptor tyrosine kinase inhibitor (EGFR-TKI) is widely used for treatment of non-small cell lung cancer (NSCLC) patients with *EGFR* mutation. However, intracranial metastasis and progression could happen to some patients while their primary tumors in lung stabilize or even shrink after EGFR-TKI treatment. And mechanisms on this phenomenon remain unclear. Recently, increasing studies have demonstrated that it is associated with the pharmacokinetics of EGFR-TKI, heterogeneity between primary tumors and metastases, *EGFR* mutation status and prolonged survival. Therefore, we write this review to discuss the mechanisms on intracranial metastasis of NSCLC after clinical benefit from EGFR-TKI.

肺癌是目前全球发病率和病死率最高的恶性肿瘤之一，其中非小细胞肺癌（non-small cell lung cancer, NSCLC）约占所有肺癌的85%^[1]^。目前，表皮生长因子受体酪氨酸激酶抑制剂（epidermal growth factor receptor-tyrosine kinase inhibitor, EGFR-TKI）已成为*EGFR*突变阳性NSCLC患者的首选治疗，并使这些患者的中位生存期提高至35个月^[2, 3]^。多项临床研究^[4-6]^表明，EGFR-TKI治疗包括吉非替尼、厄洛替尼、埃可替尼等均能有效延长患者的无进展生存期。然而，尽管EGFR-TKI的应用使58.0%-82.9%的*EGFR*突变阳性NSCLC患者的肺部原发病灶缩小甚至达到临床完全缓解^[7]^，仍有部分患者出现中枢神经系统（central nervous system, CNS）的转移或原有颅内转移灶的进展^[8, 9]^。因此，NSCLC患者接受EGFR-TKI治疗并获益后发生颅内转移的相关机制已成为近年来的研究热点。

## 1 NSCLC在EGFR-TKI治疗获益后颅内转移现状

目前研究^[10]^显示，即使在肺部原发病灶得到控制、整体生存期延长的情况下，NSCLC患者在EGFR-TKI治疗过程中出现新发颅内转移或原有颅内转移灶进展的事件仍频繁发生。Lee等^[11]^对287例韩国腺癌患者进行的队列研究显示，在166例对EGFR-TKI治疗临床获益的患者中，26%的患者在肺部原发灶得到临床控制的情况下发生了CNS转移（包括脑实质和软脑膜），而13%的患者发生了孤立性的CNS进展，这也是其主要的致死原因。Omuro等^[8]^对139例接受吉非替尼标准治疗的NSCLC患者进行评估，其结果表明在21例肺内癌灶得到部分缓解的患者中，仍有7例（33%）出现了新发的CNS转移灶，其中5例为脑实质转移，2例为软脑膜转移。此外，北京协和医院肺癌中心的一项大型研究^[9]^也发现，在316例*EGFR*突变阳性的晚期NSCLC患者中，51例（16.14%）在接受吉非替尼靶向治疗的情况下发生了CNS复发，仅次于肺转移与骨转移。值得说明的是，由于回顾性研究的内在缺陷，上述研究得到的颅内转移发生率还应低于实际的数据。由此可见，NSCLC的肺部原发灶与颅内转移灶对EGFR-TKI存在着不同的治疗反应^[12]^，而患者机体对EGFR-TKI的耐药性无法完全解释这种现象的存在。

## 2 NSCLC在EGFR-TKI治疗获益后颅内转移的相关机制研究

### 2.1 药物代谢动力学

中枢神经系统是EGFR-TKI治疗有效后NSCLC转移与复发的高发场所，而EGFR-TKI不完全的血脑屏障通过率是目前最为广泛接受的原因。化疗药物的血脑屏障通过率由多种因素决定，包括其分子量、脂溶性、离子化程度、血浆蛋白结合率及该药物分子与膜转运载体的亲和力^[13]^。EGFR-TKI属于小分子化合物，其分子量一般在400 Da以上，脂溶性较强，但血浆蛋白结合率均大于90%，不易以被动扩散的方式通过血脑屏障。因此，EGFR-TKI的血脑屏障通过率主要与血脑屏障上的转运载体ATP结合盒转运体（ATP-binding cassette transporter, ABC transporter）有关。ABC转运体包括P-糖蛋白（P-glycoprotein, P-gp）、乳腺癌耐药蛋白、多药耐药相关蛋白等，其中P-gp是血脑屏障的主要膜转运蛋白，它发挥着将化疗药物与有毒物质主动泵出中枢神经系统的重要作用^[14]^。EGFR-TKI与P-gp的亲和力决定了其血脑屏障通过率与脑脊液浓度，研究^[15]^发现P-gp抑制剂（伐司扑达、依克立达等）的应用可以提高多种肿瘤化疗药物的脑脊液浓度，包括吉非替尼、厄洛替尼、顺铂、紫杉醇。同时，不同患者的血脑屏障膜转运蛋白的多态性会直接导致EGFR-TKI血脑屏障通过率的个体差异，从而影响EGFR-TKI的治疗效果^[16]^。因此，血脑屏障上多种转运载体（包括BCRP1、Abcg2等）对EGFR-TKI的外排作用机制及ABC转运体抑制剂对EGFR-TKI药代动力学的影响仍有待更深入的研究。

已有研究^[17]^证明，吉非替尼对EGFR酪氨酸激酶活性的半数抑制浓度（median inhibitory concentration 50, IC_50_）为27 nmol/L，对*EGFR*突变的肿瘤细胞生长的IC_50_为54 nmol/L，但接受吉非替尼标准治疗（250 mg/d）的患者脑脊液中的药物浓度明显低于其IC_50_，这很可能是吉非替尼的血脑屏障通过率仅为1%所致；厄洛替尼对*EGFR*突变阳性细胞系生长的IC_50_为100 nmol/L，其血脑屏障通过率为2.5%-13.0%，尽管标准治疗剂量（150 mg/d）能使其血浆浓度达到3, 000 nmol/L，但其脑脊液药物浓度仍然显著低于IC_50_^[18]^。Deng等^[19]^研究显示，已有CNS转移的6例*EGFR*突变阳性NSCLC患者在接受厄洛替尼标准治疗（150 mg/d）4周后，其血浆与脑脊液中的厄洛替尼平均浓度分别为（717.7±459.7）ng/mL、（23.7±13.4）ng/mL，其平均血脑屏障通过率仅为（4.4%±3.2%）。在一项随机对照双盲试验中^[20]^，15例NSCLC颅内转移的患者随机分组，并分别接受吉非替尼与厄洛替尼标准治疗，最终吉非替尼在脑脊液中的平均浓度与血脑屏障通过率分别为（3.7±1.9）ng/mL（1.13%±0.36%），而厄洛替尼相应的浓度与透过率分别为（28.7±16.8）ng/mL（2.77%±0.45%）。因此，对于已有CNS转移的NSCLC患者，厄洛替尼的有效率高于吉非替尼。另一方面，有学者^[21]^发现脑实质癌细胞胞浆内的EGFR-TKI药物浓度与其脑脊液浓度呈正相关（R^2^=0.84, P=0.000, 5），给予患者大剂量的EGFR-TKI治疗有助于EGFR-TKI在CNS转移灶内发挥抗癌作用。Clarke等^[18]^对*EGFR*突变阳性的NSCLC患者给予脉冲式的大剂量厄洛替尼（1, 000 mg/w-1, 200 mg/w）时发现，患者脑脊液中厄洛替尼的浓度达到了治疗浓度，颅内病灶得到较好的控制。同时，Yi等^[22]^对7例已有颅内转移的NSCLC患者予以大剂量吉非替尼（500 mg-700 mg）治疗，也得出了相似的研究结果。这也间接说明了EGFR-TKI的脑脊液浓度不足是导致NSCLC患者在肺部病灶稳定、缓解的同时发生颅内转移与进展的重要原因，适当增大EGFR-TKI给药剂量可能有利于NSCLC患者的临床治疗，但目前仍缺乏相关的大型临床研究资料。因此，EGFR-TKI的用药剂量、脑脊液浓度和NSCLC颅内转移复发事件之间的相关性仍待进一步研究，积极研发具有更高血脑屏障通过率的新一代EGFR-TKI（如阿法替尼）将对NSCLC的有效治疗有着重要的临床意义。

### 2.2 NSCLC原发灶与转移灶的异质性

研究^[23]^发现，EGFR-TKI治疗获益后NSCLC患者发生颅内转移或进展现象可能与转移癌灶的*EGFR*突变状态异质性有关。原发肿瘤与转移病灶在*EGFR*基因和转录水平存在明显的异质性，该现象在乳腺癌、胰腺癌与胶质瘤等多种模型中已得到证实^[24-26]^。包括9项临床研究、707例NSCLC患者的荟萃分析结果^[27]^显示，*EGFR*突变的NSCLC患者的原发病灶与淋巴结转移灶的*EGFR*突变状态存在12.2%的差异率，这说明*EGFR*突变状态在NSCLC的发展过程中是不断变化的。此外，也有研究^[28]^证实，*EGFR*突变状态的变化长期存在于肿瘤发生、发展的过程中，即使在同一个病灶的不同时期，其*EGFR*突变状态也存在着异质性。其中，肺内转移灶与原发灶的差异率是24.4%，而远处转移灶（包括CNS、肝、骨等）与原发灶的差异率是14.3%，180例亚裔NSCLC患者的总体差异率是13.9%。更重要的是，接受EGFR-TKI治疗后患者原发灶与转移灶的差异率高达26.3%，提示EGFR-TKI可能本身就是潜在的*EGFR*诱变因素。

近年来多项研究^[29, 30]^表明，NSCLC患者颅内转移病灶与颅外癌灶的*EGFR*突变状态确实存在差异，体现在*EGFR*的基因序列、突变类型和突变频率等多个方面。应用EGFR-TKI治疗后发生NSCLC颅内转移的患者，其CNS内的*EGFR*突变体的类型、性质均与其他部位转移灶（如肺、骨）不同^[31]^。Daniele等^[32]^同时获取26例已发生CNS转移患者的肺部原发灶和颅内转移灶的肿瘤组织，进行荧光原位杂交（fluorescence *in situ* hybridization，FISH）分析和直接测序，结果显示肺部原发灶的FISH阳性率为28%，而转移灶的FISH阳性率为45%；其中，6例CNS转移患者表现为转移灶FISH阳性而原发灶FISH阴性，提示颅内转移灶和肺部原发灶之间的异质性可能是导致两者对EGFR-TKI治疗产生不同反应的重要原因之一。对于NSCLC患者而言，远处转移尤其是颅内转移是其主要的致死原因。因此，根据颅内转移灶而不是肺部原发灶的*EGFR*突变状态来预测患者对EGFR-TKI靶向治疗的临床反应更具临床意义^[33]^。

### 2.3 *EGFR*突变本身特质

对于晚期NSCLC患者而言，*EGFR*突变是预测EGFR-TKI药物疗效的关键因素，*EGFR*突变阳性通常意味着该患者对EGFR-TKI治疗反应良好。然而，*EGFR*突变也可能导致NSCLC患者更高的颅内转移发生率。Matsumoto等^[34]^研究发现，相对于*EGFR*野生型的NSCLC患者，*EGFR*突变阳性患者更容易发生CNS转移与进展，提示*EGFR*基因突变本身可能就是NSCLC发生颅内转移的危险因子之一，在肺部癌变过程中早期产生的*EGFR*突变可能增加NSCLC的颅内转移趋势。

Li等^[35]^通过对110例晚期NSCLC患者回顾性分析发现，相对于未发生CNS转移的患者，已发生CNS转移患者的*EGFR*基因突变更为频繁。进一步研究表明，与野生型*EGFR*患者比较，*EGFR*突变阳性患者在手术切除原发病灶后的总体复发率稍低，但CNS复发事件却更加常见（9% *vs* 24%, *P* < 0.015）^[36]^。Eichler等^[37]^也发现，*EGFR*突变阳性的NSCLC患者比野生型*EGFR*患者更易于发生CNS转移。一项对283例中国肺腺癌患者的回顾性研究也得到类似发现，*EGFR*突变频率与患者胸膜转移的发病率呈正相关，这也提示*EGFR*突变本身特质对NSCLC癌细胞的生物学行为存在一定影响^[38]^，但是其中的具体机制仍待更深入的研究。

### 2.4 患者生存期的延长

已有多项临床研究证明，EGFR-TKI治疗能延长NSCLC患者的无进展生存期和总生存期，并提高其生活质量。因此，一般的回顾性研究认为，颅内转移与进展作为NSCLC病程中的晚期事件和主要致死原因之一，患者生存期的延长显然会导致其颅内转移事件的统计学数据相应增高。Lee等^[11]^的队列研究表明，相较于对EGFR-TKI无临床获益的NSCLC患者，EGFR-TKI临床获益患者的疾病进展时间（8.5个月*vs* 1.7个月，*P* < 0.001）、总生存期（24.5个月*vs* 4.3个月，*P* < 0.001）及疾病进展时间（19.4个月*vs* 10.6个月，*P* < 0.001）均明显延长。进一步的*Logistic*单变量分析结果显示，EGFR-TKI治疗临床获益反应与疾病进展时间的延长均可使NSCLC患者发生孤立CNS转移的风险显著增加，其风险比分别为15.8（*P* < 0.001）、1.04（*P* < 0.008）。然而，多变量分析却提示，仅有EGFR-TKI药物的临床获益反应是孤立CNS转移与进展的独立预测因子（调整风险比为10.9，*P*=0.01）。因此，疾病进展时间或患者总生存期的延长并不能单独解释晚期NSCLC患者CNS转移与进展的发生，仍必需结合前述的多个相关因素进行分析，但生存期的延长会放大这些因素的影响^[39]^。

## 3 结语

综上所述，NSCLC患者对EGFR-TKI治疗临床获益后发生颅内转移与进展是临床常见现象，也是严重威胁NSCLC患者生存的重要危险因素。近年来的研究指出，该现象主要与EGFR-TKI的药物代谢动力学、NSCLC原发灶与转移灶的异质性及*EGFR*突变本身特质有关，而EGFR-TKI治疗后患者生存期的延长则会导致统计的颅内转移事件相应地增多。但目前的研究资料尚不足以完全阐明相关机制，也无法对NSCLC颅内转移后的治疗方案提供有力的指导。因此，如何针对上述可能的机制和靶点，进一步研发有效的临床药物与治疗措施，在控制肺内原发病灶的同时尽量减少甚至避免颅内转移与进展事件的发生，将是一项艰巨的任务。

## References

[1] Li N, Yang L, Ou W (2014). *Meta*-analysis of EGFR tyrosine kinase inhibitors compared with chemotherapy as second-line treatment in pretreated advanced non-small cell lung cancer. PLoS One.

[2] Chen YM (2013). Update of epidermal growth factor receptor-tyrosine kinase inhibitors in non-small-cell lung cancer. J Chin Med Assoc.

[3] Cufer T, Knez L (2014). Update on systemic therapy of advanced non-small-cell lung cancer. Expert Rev Anticancer Ther.

[4] Shepherd FA, Rodrigues PJ, Ciuleanu T (2005). Erlotinib in previously treated non-small-cell lung cancer. N Engl J Med.

[5] Zhao Q, Shentu J, Xu N (2011). Phase I study of icotinib hydrochloride (BPI-2009H), an oral EGFR tyrosine kinase inhibitor, in patients with advanced NSCLC and other solid tumors. Lung Cancer.

[6] Melosky B (2014). Review of EGFR TKIs in metastatic NSCLC, including ongoing trials. Front Oncol.

[7] Peng L, Song ZG, Jiao SC (2014). Efficacy analysis of tyrosine kinase inhibitors on rare non-small cell lung cancer patients harboring complex *EGFR* mutations. Sci Rep.

[8] Omuro AM, Kris MG, Miller VA (2005). High incidence of disease recurrence in the brain and leptomeninges in patients with nonsmall cell lung carcinoma after response to gefitinib. Cancer.

[9] Chen MJ, Zhong W, Zhang L (2013). Recurrence patterns of advanced non-small cell lung cancer treated with gefitinib. Chin Med J (Engl).

[10] Burel-Vandenbos F, Ambrosetti D, Coutts M (2013). *EGFR* mutation status in brain metastases of non-small cell lung carcinoma. J Neurooncol.

[11] Lee YJ, Choi HJ, Kim SK (2010). Frequent central nervous system failure after clinical benefit with epidermal growth factor receptor tyrosine kinase inhibitors in Korean patients with nonsmall-cell lung cancer. Cancer.

[12] Schuler M, Fischer JR, Grohe C (2014). Experience with afatinib in patients with non-small cell lung cancer progressing after clinical benefit from gefitinib and erlotinib. Oncologist.

[13] Kemper EM, Boogerd W, Thuis I (2004). Modulation of the blood-brain barrier in oncology: therapeutic opportunities for the treatment of brain tumours?. Cancer Treat Rev.

[14] Elmeliegy MA, Carcaboso AM, Tagen M (2011). Role of ATP-binding cassette and solute carrier transporters in erlotinib CNS penetration and intracellular accumulation. Clin Cancer Res.

[15] Noguchi K, Kawahara H, Kaji A (2009). Substrate-dependent bidirectional modulation of P-glycoprotein-mediated drug resistance by erlotinib. Cancer Sci.

[16] Jamal-Hanjani M, Spicer J (2012). Epidermal growth factor receptor tyrosine kinase inhibitors in the treatment of epidermal growth factor receptor-mutant non-small cell lung cancer metastatic to the brain. Clin Cancer Res.

[17] Mckillop D, Partridge EA, Kemp JV (2005). Tumor penetration of gefitinib (Iressa), an epidermal growth factor receptor tyrosine kinase inhibitor. Mol Cancer Ther.

[18] Clarke JL, Pao W, Wu N (2010). High dose weekly erlotinib achieves therapeutic concentrations in CSF and is effective in leptomeningeal metastases from epidermal growth factor receptor mutant lung cancer. J Neurooncol.

[19] Deng Y, Feng W, Wu J (2014). The concentration of erlotinib in the cerebrospinal fluid of patients with brain metastasis from non-small-cell lung cancer. Mol Clin Oncol.

[20] Togashi Y, Masago K, Masuda S (2012). Cerebrospinal fluid concentration of gefitinib and erlotinib in patients with non-small cell lung cancer. Cancer Chemother Pharmacol.

[21] Togashi Y, Masago K, Fukudo M (2011). Efficacy of increased-dose erlotinib for central nervous system metastases in non-small cell lung cancer patients with epidermal growth factor receptor mutation. Cancer Chemother Pharmacol.

[22] Yi HG, Kim HJ, Kim YJ (2009). Epidermal growth factor receptor (EGFR) tyrosine kinase inhibitors (TKIs) are effective for leptomeningeal metastasis from non-small cell lung cancer patients with sensitive *EGFR* mutation or other predictive factors of good response for EGFR TKI. Lung Cancer.

[23] Berger LA, Riesenberg H, Bokemeyer C (2013). CNS metastases in non-small-cell lung cancer: current role of EGFR-TKI therapy and future perspectives. Lung Cancer.

[24] Weigelt B, Ng CK, Shen R (2015). Metastatic breast carcinomas display genomic and transcriptomic heterogeneity. Mod Pathol.

[25] Graham JS, Jamieson NB, Rulach R (2015). Pancreatic cancer genomics: where can the science take us?. Clin Genet.

[26] D'Asti E, Fang Y, Rak J (2014). Brain neoplasms and coagulation-lessons from heterogeneity. Rambam Maimonides Med J.

[27] Wang F, Fang P, Hou DY (2014). Comparison of epidermal growth factor receptor mutations between primary tumors and lymph nodes in non-small cell lung cancer: a review and *meta*-analysis of published data. Asian Pac J Cancer Prev.

[28] Chen ZY, Zhong WZ, Zhang XC (2012). *EGFR* mutation heterogeneity and the mixed response to EGFR tyrosine kinase inhibitors of lung adenocarcinomas. Oncologist.

[29] Jackman DM, Holmes AJ, Lindeman N (2006). Response and resistance in a non-small-cell lung cancer patient with an epidermal growth factor receptor mutation and leptomeningeal metastases treated with high-dose gefitinib. J Clin Oncol.

[30] Ruppert AM, Beau-Faller M, Neuville A (2009). EGFR-TKI and lung adenocarcinoma with CNS relapse: interest of molecular follow-up. Eur Respir J.

[31] Balak MN, Gong Y, Riely GJ (2006). Novel D761Y and common secondary T790M mutations in epidermal growth factor receptor-mutant lung adenocarcinomas with acquired resistance to kinase inhibitors. Clin Cancer Res.

[32] Daniele L, Cassoni P, Bacillo E (2009). Epidermal growth factor receptor gene in primary tumor and metastatic sites from non-small cell lung cancer. J Thorac Oncol.

[33] Sun M, Behrens C, Feng L (2009). HER family receptor abnormalities in lung cancer brain metastases and corresponding primary tumors. Clin Cancer Res.

[34] Matsumoto S, Takahashi K, Iwakawa R (2006). Frequent *EGFR* mutations in brain metastases of lung adenocarcinoma. Int J Cancer.

[35] Li Z, Lu J, Zhao Y, Gou H (2011). The retrospective analysis of the frequency of *EGFR* mutations and the efficacy of gefitinib in NSCLC patients with brain metastasis. J Clin Oncol.

[36] Lee YJ, Park IK, Park MS (2009). Activating mutations within the EGFR kinase domain: a molecular predictor of disease-free survival in resected pulmonary adenocarcinoma. J Cancer Res Clin Oncol.

[37] Eichler AF, Kahle KT, Wang DL (2010). *EGFR* mutation status and survival after diagnosis of brain metastasis in non small cell lung cancer. Neuro Oncol.

[38] Zou J, Bella A E, Chen Z (2014). Frequency of *EGFR* mutations in lung adenocarcinoma with malignant pleural effusion: Implication of cancer biological behaviour regulated by *EGFR* mutation. J Int Med Res.

[39] Kim HR, Lee JC, Kim YC (2014). Clinical characteristics of non-small cell lung cancer patients who experienced acquired resistance during gefitinib treatment. Lung Cancer.

